# 3D Whole Heart Imaging for Congenital Heart Disease

**DOI:** 10.3389/fped.2017.00036

**Published:** 2017-02-27

**Authors:** Gerald Greil, Animesh (Aashoo) Tandon, Miguel Silva Vieira, Tarique Hussain

**Affiliations:** ^1^Department of Pediatrics, University of Texas Southwestern Medical Center, Dallas, TX, USA; ^2^Department of Radiology, University of Texas Southwestern Medical Center, Dallas, TX, USA; ^3^Department of Biomedical Engineering, University of Texas Southwestern Medical Center, Dallas, TX, USA; ^4^Division of Pediatric Cardiology, Children’s Medical Center Dallas, Dallas, TX, USA; ^5^Division of Imaging Sciences and Biomedical Engineering, King’s College London, London, UK

**Keywords:** three-dimensional whole heart imaging, congenital heart disease, cardiovascular MRI, pediatrics, coronary imaging

## Abstract

Three-dimensional (3D) whole heart techniques form a cornerstone in cardiovascular magnetic resonance imaging of congenital heart disease (CHD). It offers significant advantages over other CHD imaging modalities and techniques: no ionizing radiation; ability to be run free-breathing; ECG-gated dual-phase imaging for accurate measurements and tissue properties estimation; and higher signal-to-noise ratio and isotropic voxel resolution for multiplanar reformatting assessment. However, there are limitations, such as potentially long acquisition times with image quality degradation. Recent advances in and current applications of 3D whole heart imaging in CHD are detailed, as well as future directions.

## Introduction

The three-dimensional (3D) whole heart approach with respiratory navigator gating and ECG triggering has been developed to enable coronary imaging (1). This free-breathing and radiation-free approach is well established for the detection of coronary artery anomalies in infants and young children with congenital heart disease (CHD) (2) but is less used for assessment of coronary stenoses in adults (3). The comprehensive evaluation of thoracic vasculature it offers is uniquely suited to give detailed morphological information in CHD. There are a number of developments, mostly related to improved motion correction, which have made this approach feasible. Early reports of coronary imaging used multiple breath-holds and set the cardiac motion for diastole by using the estimated percentage of the RR interval (4); however, this approach yielded images of suboptimal quality. Important developments were then made in this regard: first, work by Kim et al. showed that improved image quality was obtained by individually defining the cardiac rest periods (5); second, advances in image contrast improved overall image quality (6); and finally, respiratory motion was addressed through the use of navigators during free-breathing coronary MR (7).

## Rest Periods

Cardiac rest periods for imaging include mid-diastole (between the early and rapid filling periods of the left ventricle) and end-systole (between aortic valve closure and mitral valve opening). In order to “freeze” coronary artery motion and minimize image blurring, the longest rest period with the least cardiac motion is often chosen, which is usually in mid-diastole. The longer rest period allows more data to be acquired per heartbeat to fill k-space. End-diastole can be reasonably estimated for the majority of patients using a trigger time that starts at approximately 75% of the RR interval (8).

Using a “one-size-fits-all” approach, however, has been shown to result in inferior image quality (5). This is more critical in MRI compared to computer tomography (CT) coronary imaging owing to the greater flexibility in defining the acquisition window and data reconstruction over multiple cardiac cycles, and is particularly true for children, for whom the heart rate and RR interval and respiratory pattern variability is often high. In fact, as the heart rate increases, the mid-diastolic rest period shortens significantly. With this in mind, Tangcharoen et al. showed that prospective selection of end-systole over end-diastole greatly improved the success in implementing the whole heart sequence in children (9). Using this approach, they were able to demonstrate coronary origins (as confirmed by surgery) in greater than 88% of children above 4 months of age. However, success below this age has continued to be elusive and experience limited.

**Figure 1:**
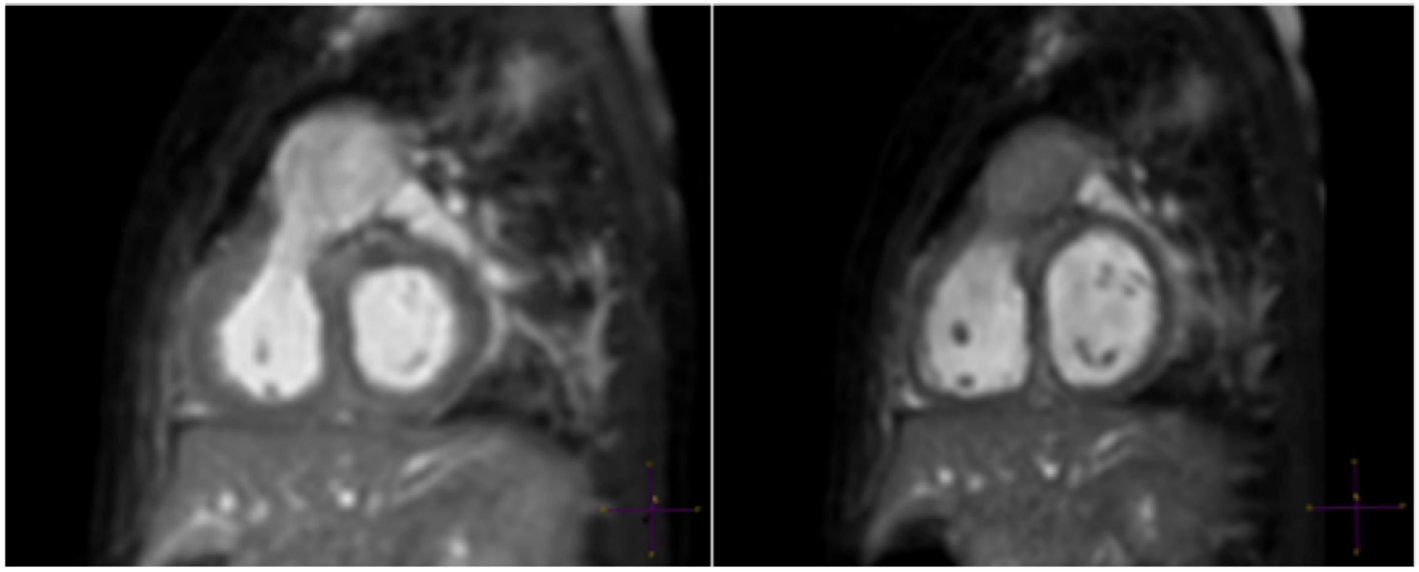
**A 6-month-old patient with right ventricular outflow tract (RVOT) aneurysm**. “Windowing” levels and geometry are linked in this multiplanar reformat of a RVOT aneurysm showing clear superiority of the systolic image (left).

The conundrum regarding the optimal phase for imaging was eventually solved by Uribe et al. by developing a dual-phase sequence that was capable of acquiring both rest periods in a single acquisition with a similar imaging time to a single phase sequence (10). The dual-phase sequence is noteworthy for several reasons. First, it became apparent that although the end-systolic rest period is longer at higher heart rates, some coronary segments, such as the right coronary artery (RCA) within the anterior atrioventricular groove, may still be seen better during mid-diastole. Given this finding, the ability to perform dual-phase imaging with prospective selection of trigger delays for systole and diastole and with retrospective selection of the best phase to depict the coronary segment of interest in all cases appears attractive (10). Using this approach, Hussain et al. noted a number of advantages for CHD imaging, foremost of which was the ability to accurately measure cardiac structures in both phases (11). This is particularly important in gauging tissue properties such as distensibility prior to intervention. Furthermore, as some structures (such as pulmonary veins and atria) are better imaged in systole and others (such as great arteries and post-stenotic areas) in diastole, the dual-phase approach was shown to improve the overall success rate of imaging in CHD (Figure 1) (11). The dual-phase acquisition can also be manipulated to measure end-diastole and end-systole, which gives the imaging the ability to define ventricular volumes and ejection fraction. Although no regional wall motion function is provided, this approach allows an isotropic 3D dataset to be acquired without the necessity for breath-holding, potentially allowing for a more accurate approach to ventricular volumetric analysis (12, 13). Radial phase encoding trajectories have been also used for faster data acquisition (14).

Traditionally, the cardiac rest periods are assessed using high temporal resolution 4-chamber cine (e.g., 60–80 cardiac phases per heart beat). The mid-diastolic period is taken from cessation of movement of the RCA (i.e., pause in visible filling of RV) to the beginning of atrial systole. This stringent definition covers both RCA and left anterior descending diastolic rest periods (5). The end-systolic period is taken from cessation of movement of the RCA (corresponding to lowest RV volume) to just before the beginning of opening of the tricuspid valve. However, this method can be time-consuming and prone to interobserver error, and it has been shown that an automated program is capable of more accurate definitions of cardiac rest periods than visual inspection (15).

## Image Contrast

The importance of considering tissue boundaries for the whole heart approach was noted early on (7). Botnar et al. showed that the addition of a T2-preparation pulse resulted in relative suppression of myocardial signal and an improvement in image quality. They noted a 33% improvement in vessel sharpness and 123% improvement in contrast-to-noise ratio (CNR).

**Figure 2:**
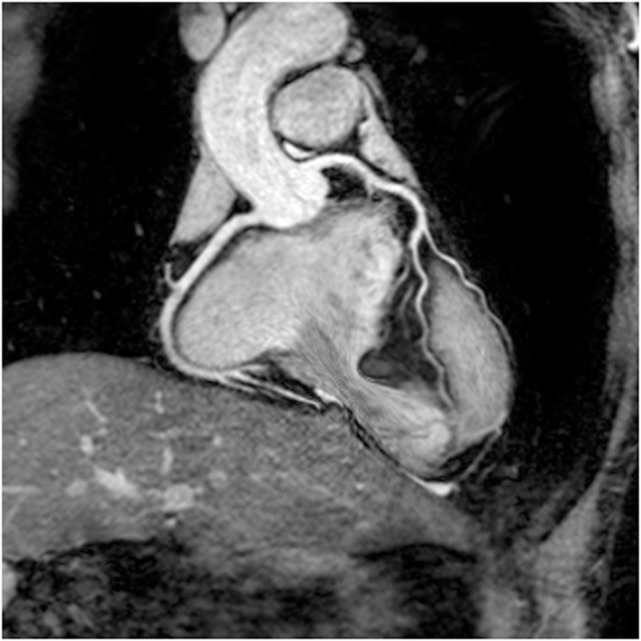
**Example of Dixon water–fat separation technique**. Reformatted coronary images demonstrating quality and superior fat suppression achieved using Dixon technique at 3 T. Courtesy of Rene Botnar and Markus Henningsson, King’s College London, London, UK.

Another important aspect of image contrast is to suppress the signal from epicardial fat (16). Fat suppression techniques that have been proposed for whole heart angiography include short tau inversion recovery and spectral presaturation with inversion recovery (SPIR). SPIR has a slight advantage in terms of tissue specificity for this purpose and results in a higher signal-to-noise ratio. Therefore, it is currently the preferred choice for fat suppression in whole heart imaging. Both methods suffer from potentially introducing misregistration artifacts or field inhomogeneity. An increasingly used method for fat suppression, the Dixon technique, relies on the phase shifts that occur due to resonance frequency differences between water and fat by acquiring images at carefully chosen echo times. This technique has been shown to give improved imaging at 1.5-T field strength (17, 18). Field homogeneity is difficult to achieve for whole heart coronary imaging at higher field strengths. This has a major impact on image quality. Therefore, the Dixon technique may offer even greater superiority over the traditional fat suppression techniques at 3.0 T (Figure 2) (17, 18).

For CHD imaging, fluid in the pericardial recesses can also cause interference with the diagnostic quality. One approach to overcome this is the use of an inversion pulse to reduce the signal from long T1 species. In addition, the null point can be set at or around the myocardium to reduce myocardial signal and obviate the need for a T2-preparation pulse. This requires shortening of the T1 time of blood by injection of a gadolinium-based contrast agent. Given the risk of contrast washout with a long acquisition time for the whole heart sequence, blood pool contrast agents have been used. Makowski et al. showed that the use of gadofosveset trisodium in combination with an inversion recovery steady-state-free-precession (SSFP) whole heart sequence was able to improve diagnostic quality and accuracy on CHD cases compared with standard extracellular contrast agents (19).

**Figure 3:**
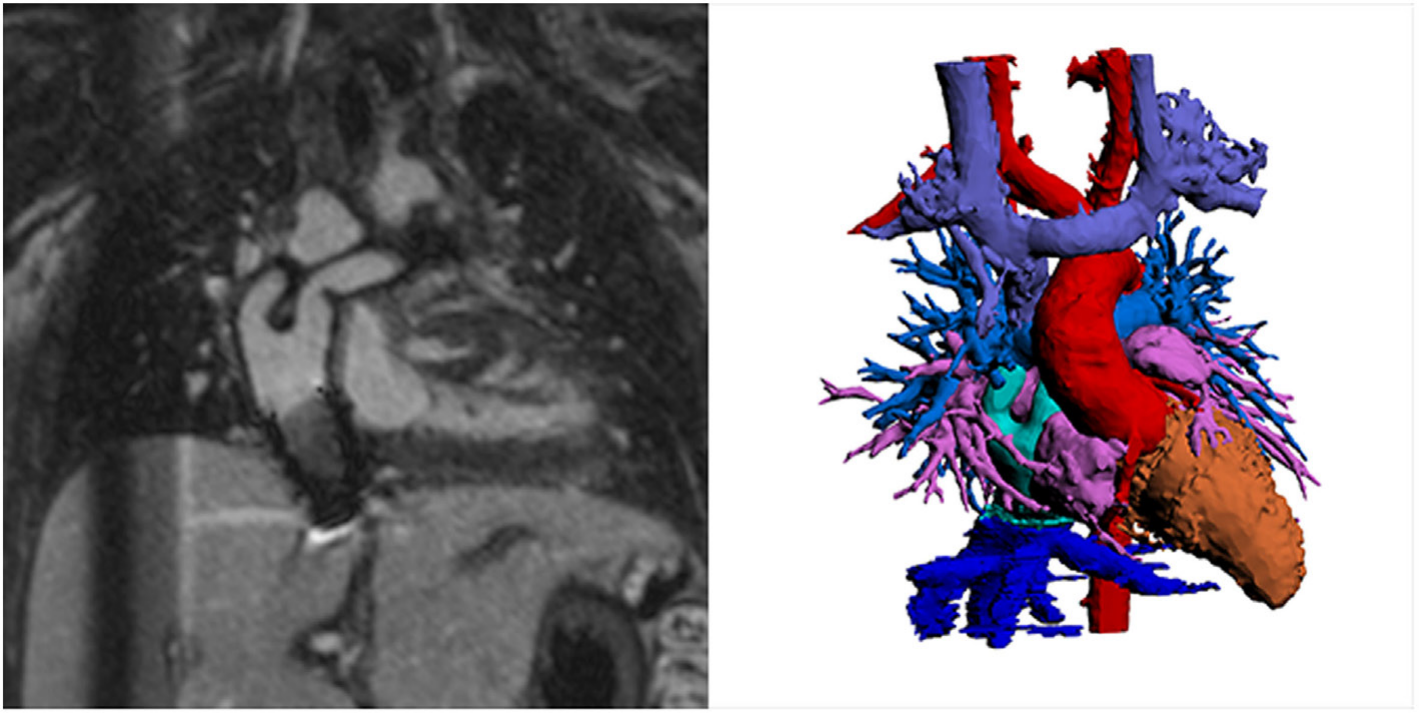
**Example inversion recovery three-dimensional spoiled gradient echo using gadofosveset trisodium image showing novel Y-graft cavopulmonary connection and inferior vena cava stent**. Spoiled gradient echo techniques show less susceptibility artifact and may be preferable for this reason. The left hand image shows source images, and the volume-rendered segmentation is shown on the right. Courtesy Tim Slesnick, MD, Children’s Healthcare of Atlanta/Emory University.

From a clinical standpoint, 3D inversion recovery SSFP imaging approach has produced the most reliable image quality, improving the ease of generating 3D models for computational simulation or 3D printing (Figure 3; Video S1 in Supplementary Material). There has been some debate as to whether this 3D whole heart inversion recovery is better with SSFP, which may give more signal, or with a spoiled gradient echo sequence, which may give greater T1-weighting allowing for more effective contrast in the presence of a blood pool gadolinium chelate. This was evaluated by Febbo et al. who concluded that SSFP was a superior approach at 1.5 T (20). However, there may be certain advantages of using a spoiled gradient echo approach, namely in terms of reduced susceptibility artifacts with metallic implants (21). Furthermore, at 3 T, SSFP sequences suffer from artifacts due to the greater field inhomogeneity at this field strength. Not surprisingly, spoiled gradient echo 3D whole heart has been shown to be superior at 3 T (Video S2 in Supplementary Material) (22).

**Figure 4:**
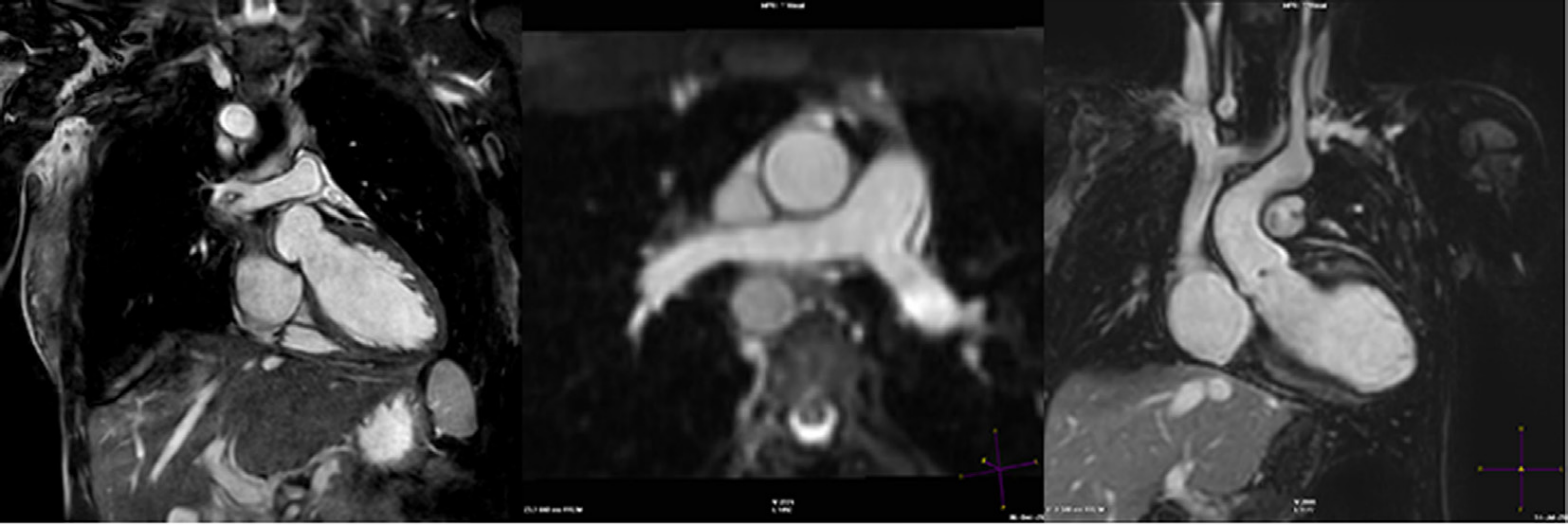
**Example images produced with gadobutrol slow infusion technique**. Image quality for slow infusion protocol with inversion recovery steady-state-free-precession three-dimensional whole heart imaging can be excellent. The inversion pulse removes signal from fluid in the pericardial recesses resulting in superior vessel sharpness.

Although providing excellent image quality, there are two problems with the inversion recovery whole heart and blood pool agent approach. First, myocardial late enhancement imaging is not possible, and second, there are currently no intravascular blood pool agents being manufactured. One possible strategy is to use gadobenate dimeglumine, which has been shown to produce similar images as gadofosveset (23) given its partial albumin-binding characteristics. However, given its linear nature, there are theoretical concerns regarding a higher risk of central nervous system deposition (24) and nephrogenic systemic fibrosis than with macrocyclic gadolinium compounds (25). For this reason, Tandon et al. recently described a practical approach to routinely using a gadobutrol slow infusion for implementation of the whole heart inversion recovery sequence. Gadobutrol is a widely used extracellular contrast agent that is macrocyclic. By administering it by slow infusion, Tandon et al. showed the ability to use this agent for inversion recovery whole heart imaging (Figure 4). Moreover, they demonstrated that it can be simultaneously used for myocardial late enhancement (26).

## Respiratory Motion

If the move from multiple breath-holds to free-breathing techniques has opened the way for higher spatial resolution coronary magnetic resonance angiography, “freezing” cardiac and coronary motion has resulted in relatively long 3D whole heart MRI acquisition times while providing sharp images (6). Besides the motion artifacts induced by cardiac pump activity and pulsatile arterial flow patterns, respiratory motion is also inevitable in free-breathing imaging techniques.

The first step toward correcting motion is to be able to measure it. This is commonly achieved by means of a respiratory navigator. This is a real-time image acquisition, which is interleaved with the high-resolution whole heart sequence, providing snapshots of the respiratory position before or after each segmented whole heart k-space acquisition. The vast majority of motion occurs in a foot–head direction, but important motion can occur in the anteroposterior and left–right directions (27). The most widely used approach for whole heart imaging is a one-dimensional diaphragmatic (1D) navigator (28). This consists of a narrow excitation pulse, typically placed at the dome of the right hemi-diaphragm, measuring the foot–head motion using a 1D representation of the lung–liver interface. However, this approach does not estimate true heart displacement, as the foot–head motion of the diaphragm is greater than the heart foot–head motion. Therefore, a correction factor of 0.6 is used to account for this. There are two problems with this estimation. First, the amount of heart motion compared to the diaphragm varies from individual to individual. Second, the heart is not “rigid,” and so respiratory motion has a more complex effect on the heart causing some shear and rotation as well. In fact, such complex motion models relating diaphragmatic to heart motion exist and are known as affine motion models, but implementation on patient-specific basis is cumbersome (29). Commercially available whole heart imaging sequences use a 1D diaphragmatic navigator, which requires a separate excitation pulse, coupled to the whole heart pulse sequence. It also requires dedicated planning alongside the imaging volume.

**Figure 5:**
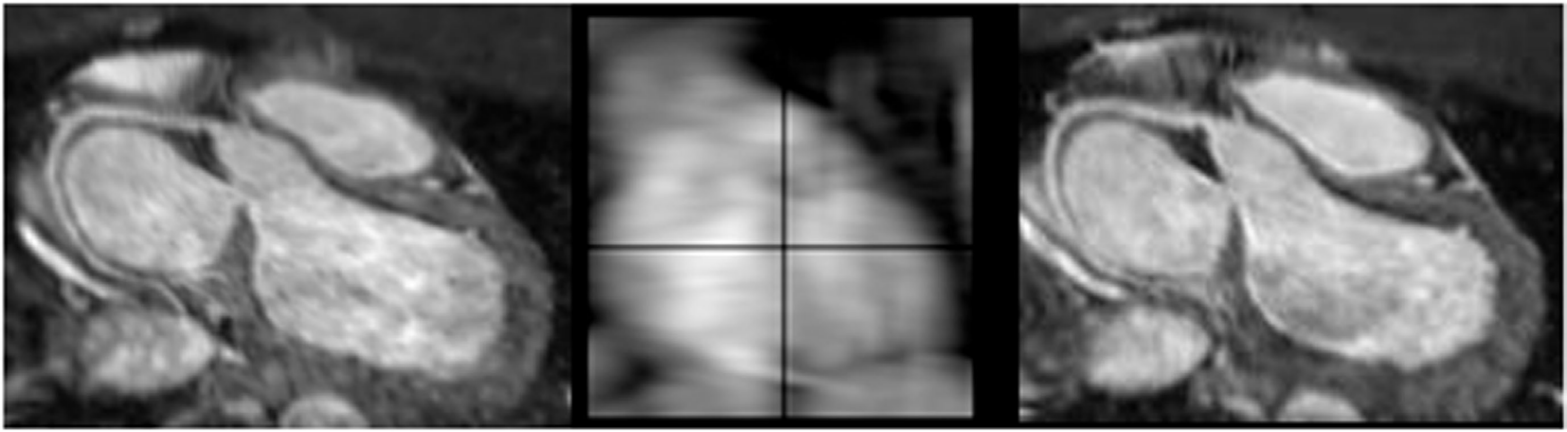
**Example images using self-navigation technique**. iNAV self-navigation using an image navigator approach depicting right coronary artery (left); sample navigator image (center); one-dimensional diaphragmatic navigator approach for comparison (right).

More recently, novel approaches have been described using simply the whole heart data itself to correct for motion. This method is known as “self-gating” (30). It has the advantage of being able to correct motion in not only the foot–head dimension, but also in all three dimensions (31). Typically, self-navigation uses a 1D projection of the FOV and so static tissue such as the chest wall is also included in the navigator image, which may interfere with the motion estimation. One method to avoid this is to confine the projection to the area of interest (e.g., the heart), using “image-based” navigation. This type of motion compensation has been applied to CHD imaging with favorable results. Henningsson et al. demonstrated that such an approach reduced scan time and improved image quality in patients with CHD compared to the convention 1D diaphragmatic navigator (32). The approach used by Henningsson et al. was further novel in that the image navigator was generated by using a low resolution 2D projection image of the heart obtained from the start-up pulses in the SSFP sequence. Hence, no further image planning or acquisition was required. Furthermore, there was no need to extend the pulse sequence design. The implementation obviated the need for dedicated navigator planning and reduced significantly the acquisition time while improving image quality. Figure 5 shows representative images showing how image-based navigation was able to depict the distal RCA. This type of image navigation is capable of correcting rigid motion in the foot–head and the left–right directions. More recently, image-based self-navigation has been implemented, which corrects for rigid and non-rigid motion in all three-dimensions (33). This type of 3D affine motion correction is currently computationally demanding, and hence difficult to implement widely.

## CHD Applications

**Figure 6:**
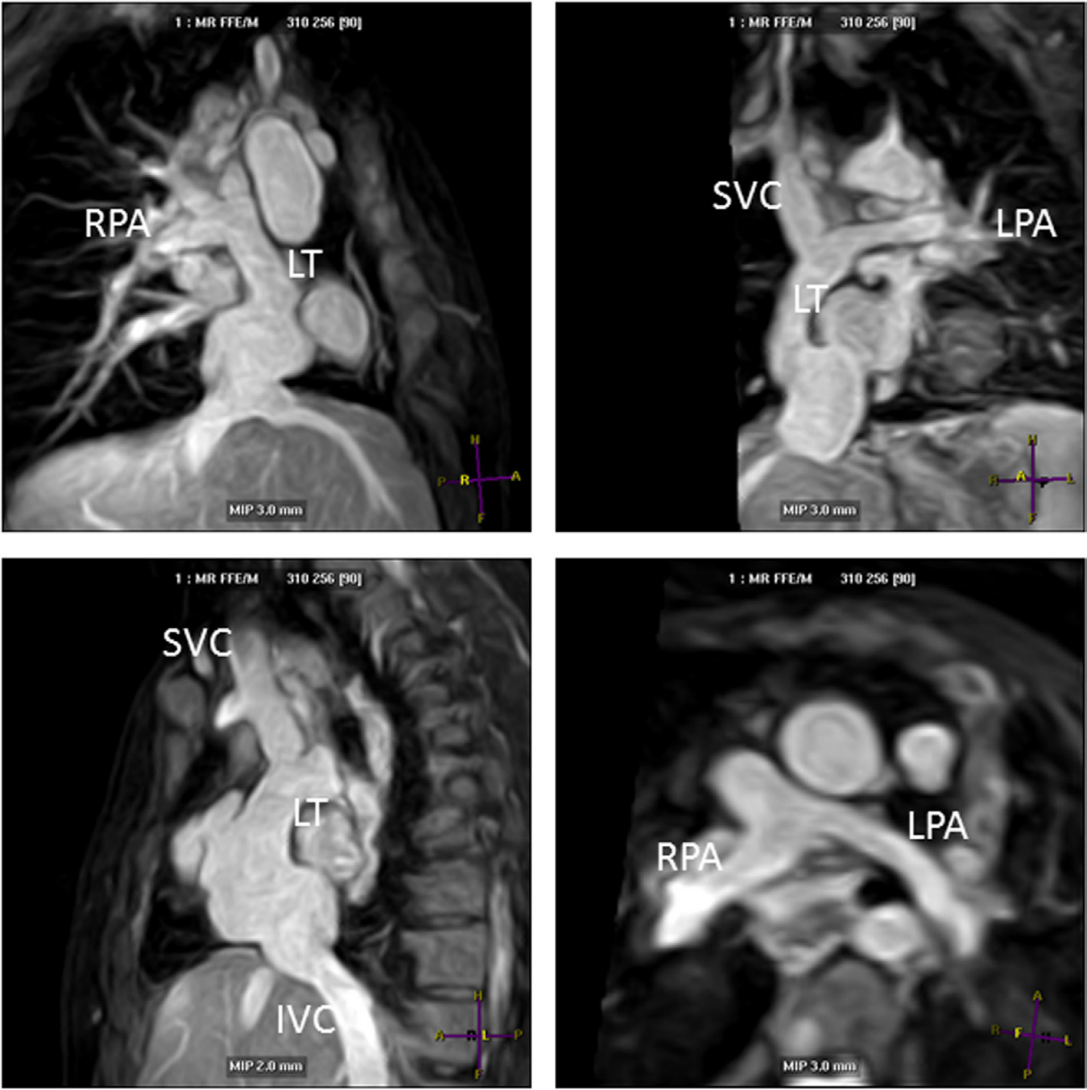
**Lateral tunnel Fontan pathways imaged using non-contrast three-dimensional (3D) balanced steady-state-free-precession (SSFP) technique**. 3D SSFP reformatted views. Abbreviations: LT, lateral tunnel; RPA, right pulmonary artery; LPA, left pulmonary artery; SVC, superior vena cava; IVC, inferior vena cava.

**Figure 7:**
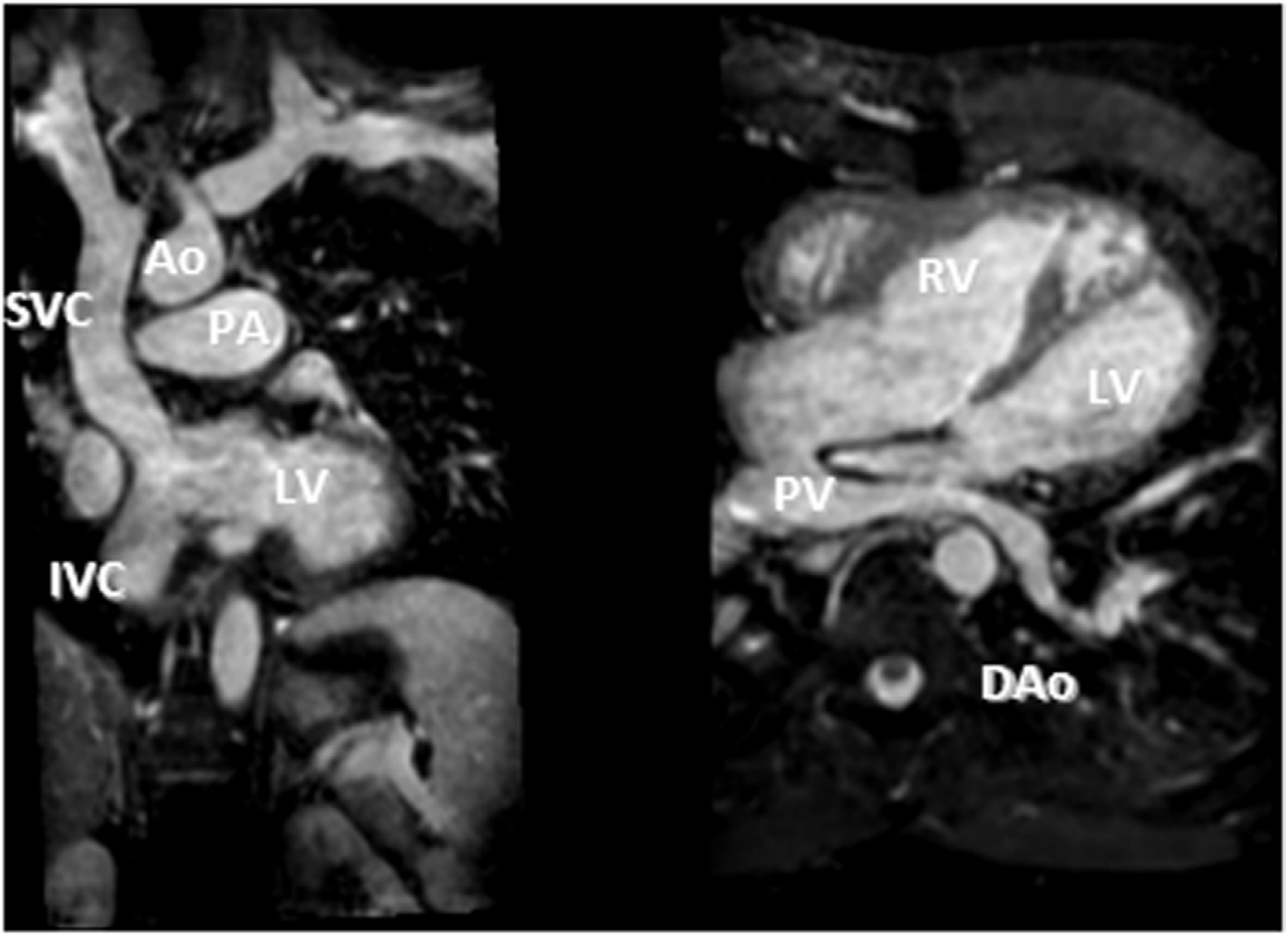
**D-TGA after atrial redirection surgery (Senning)**. Systemic venous return redirected to the left ventricle (LV), i.e., the atrial switch redirection of the inferior vena cava (IVC) and superior vena cava (SVC) flow to the LV (left). Atrial baffle redirecting pulmonary venous return to the right atrium, i.e., unobstructed pulmonary venous return, draining *via* an intracardiac tunnel at the back of the heart to the right atrium, and directed to the anatomic right ventricle (right). Also shown is how the intraatrial systemic venous Senning pathway returning to the LV is compressed in the anterior–posterior dimension.

**Figure 8:**
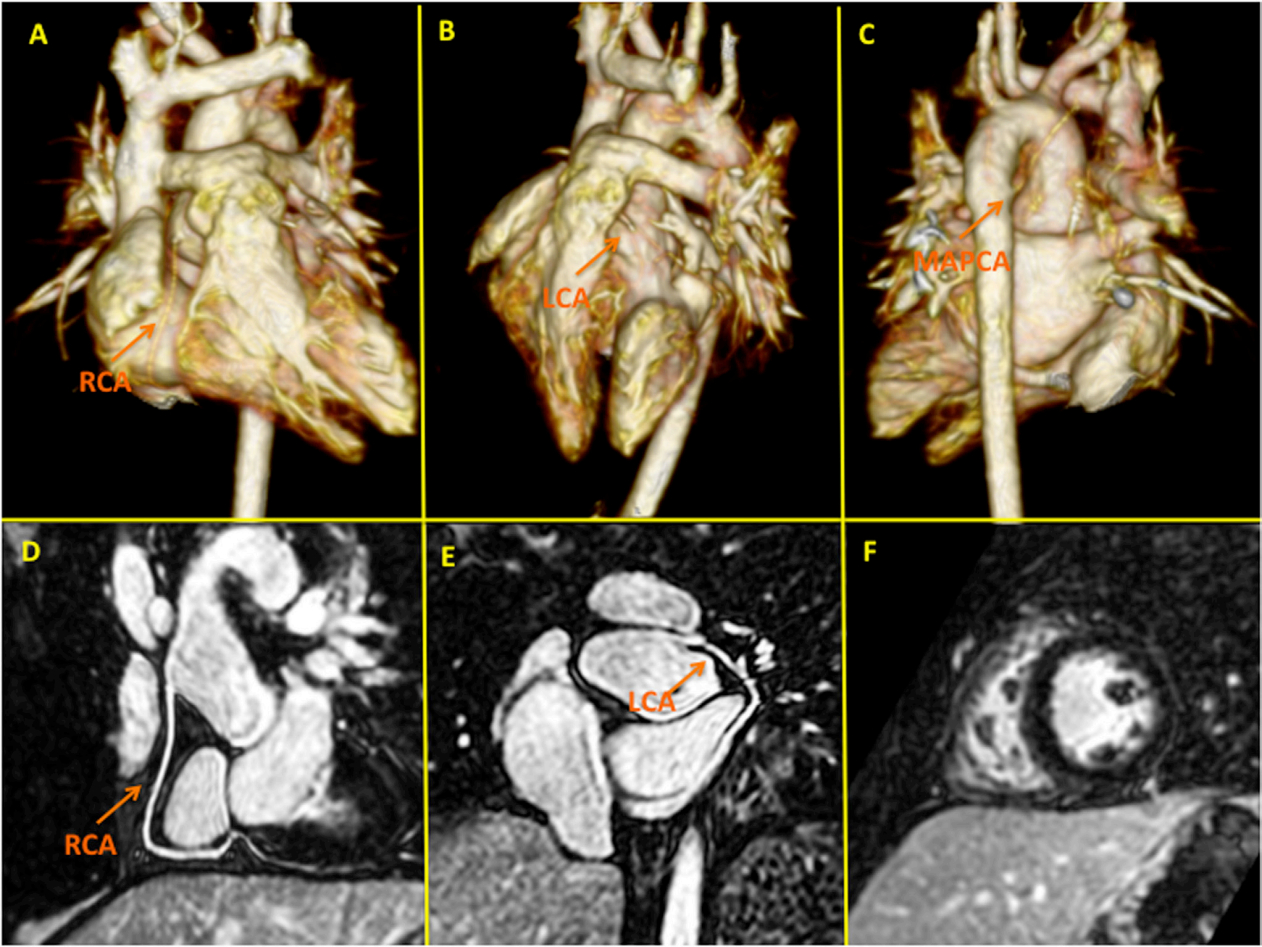
**High-spatial resolution coronary MR angiography using gadobenate dimeglumine and a novel self-navigated inversion recovery sequence in a 7-year-old patient with transposition of the great arteries**. This sequence design takes advantage of the prolonged intravascular half-life of Gd-BOPTA allowing in a single examination detailed functional **(F)**, myocardial late gadolinium enhancement, and anatomical (pulmonary, aortic, and coronary) assessment **(D,E)**. Panels **(A–C)** depict three-dimensional volume-rendered images of the pulmonary arteries following LeCompte procedure and the relation with the unobstructed coronary arteries. LCA, left coronary artery; RCA, right coronary artery; MAPCA, major aortopulmonary collateral artery.

Another major improvement in CHD MRI was the implementation of time-efficient data acquisition methods (34). These acceleration methods have allowed data acquisition of a complete 3D dataset in currently less than 5 min (26). Currently, parallel imaging is the most commonly used acceleration technique with an acceleration factor of 2 (34). This means that the sequence can be routinely clinically applied in a CHD MRI study. The acquisition of a complete 3D dataset of the cardiovascular structures of the chest and upper abdomen during systole and/or diastole allows assessment of all cardiac segments within one dataset (35). Accordingly, the segmental approach used to diagnose CHD can be easily applied retrospectively (35, 36). Moreover, the detailed 3D dataset can be used to plan further sequences (e.g., flows) to clarify specific questions as appropriate, during the scanning session. This makes this method user-independent to diagnose structural heart disease (35). Initially, the successful use has been demonstrated in adolescents and young adults (35), but with improved data acquisition techniques, infants of 4 months and older were successfully imaged using 3D whole heart imaging, both with the single phase (37) and the dual-phase approach (11). Importantly, magnetization preparation schemes have allowed arterial and venous structures to be assessed simultaneously in the majority of cases, regardless of the use of a contrast agent (Figure 6 shows Fontan pathways imaged without contrast agent use). This is a great advantage compared to CT, where high-quality coronary and vascular imaging is limited to the first pass of iodinated contrast and timed for a specific region of interest. Currently, CT studies are therefore targeted to a specific vascular structure such as the coronaries, the aorta, the pulmonary arteries, etc., and good venous and arterial signal acquisition requires additional expertise. Furthermore, despite dramatic reduction in ionizing radiation with current technologies, imaging tends to be limited to one phase if heart rate enables prospective gating. Figures 6–8 show the advantage of cardiac MRI, which is able to image both arterial and venous phases with high image signal and CNRs, while avoiding administration of contrast agents.

**Figure 9:**
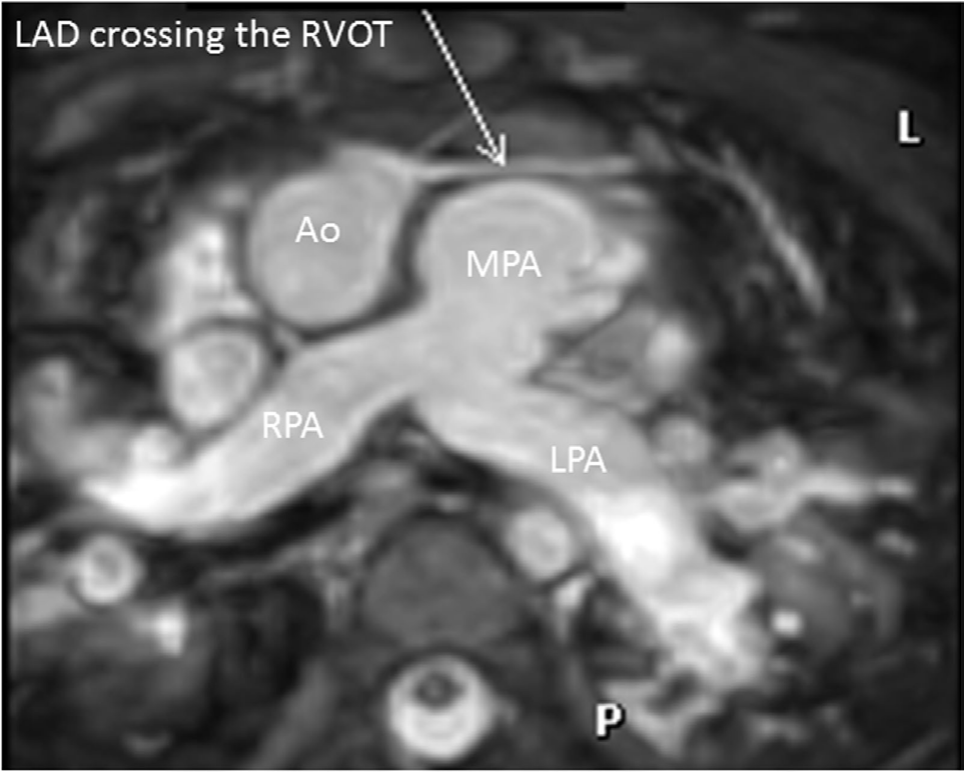
**Coronary in double outlet right ventricle**. Three-dimensional balanced steady-state-free-precession sequence demonstrating left anterior descending (LAD) artery crossing the right ventricular outflow tract (RVOT). This is an important finding to plan accurately the appropriate intervention. Abbreviations: MPA, main pulmonary artery; RPA, right pulmonary artery; LPA, left pulmonary artery.

**Figure 10:**
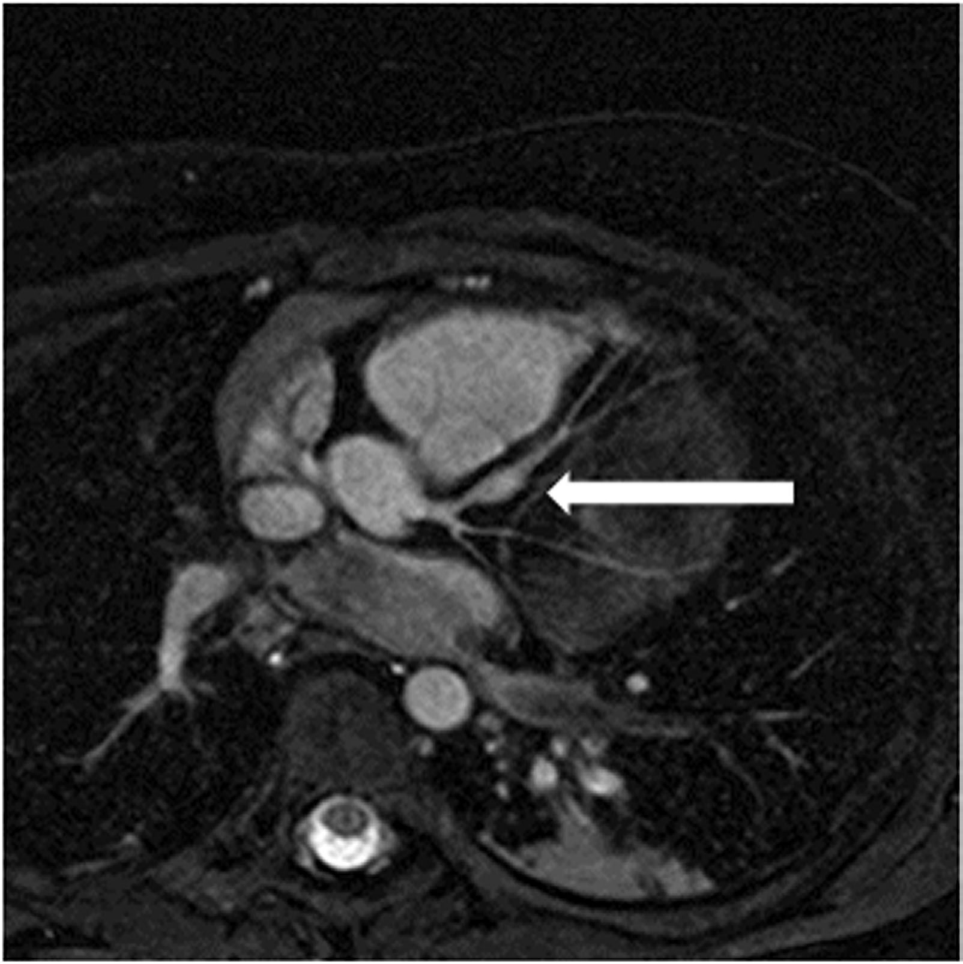
**Coronary aneurysm**. Reformatted image of a 4-year-old patient with Kawasaki disease showing aneurysm in left anterior descending coronary artery (arrow).

High quality 3D whole heart MRI datasets can display the entire lumen of the coronary arteries including their relation to neighboring structures. Accordingly, this technique has become an important and critical part of the MRI protocol for exclusion of abnormal coronary artery origins (Figure 9) (38). This is particularly important in the setting of aborted sudden cardiac death, in pediatric patients with chest pain, and in the setting of planning interventions in CHD. As described above, the advance of technology allows successful imaging of the origin and proximal course of coronary arteries in the majority of infants and young children (9). Another relevant application of this technology is the assessment of the morphology of coronary arteries in patients post-Kawasaki disease, including the location, morphology, and maximal dimensions of coronary artery aneurysms (Figure 10) (39–42). However, accurate coronary wall assessment requires specialized techniques, and unlike CT, cannot depict calcified lesions. Furthermore, other MRI techniques have shown superiority over the 3D approach described here for the assessment of coronary artery stenoses (42).

**Figure 11:**
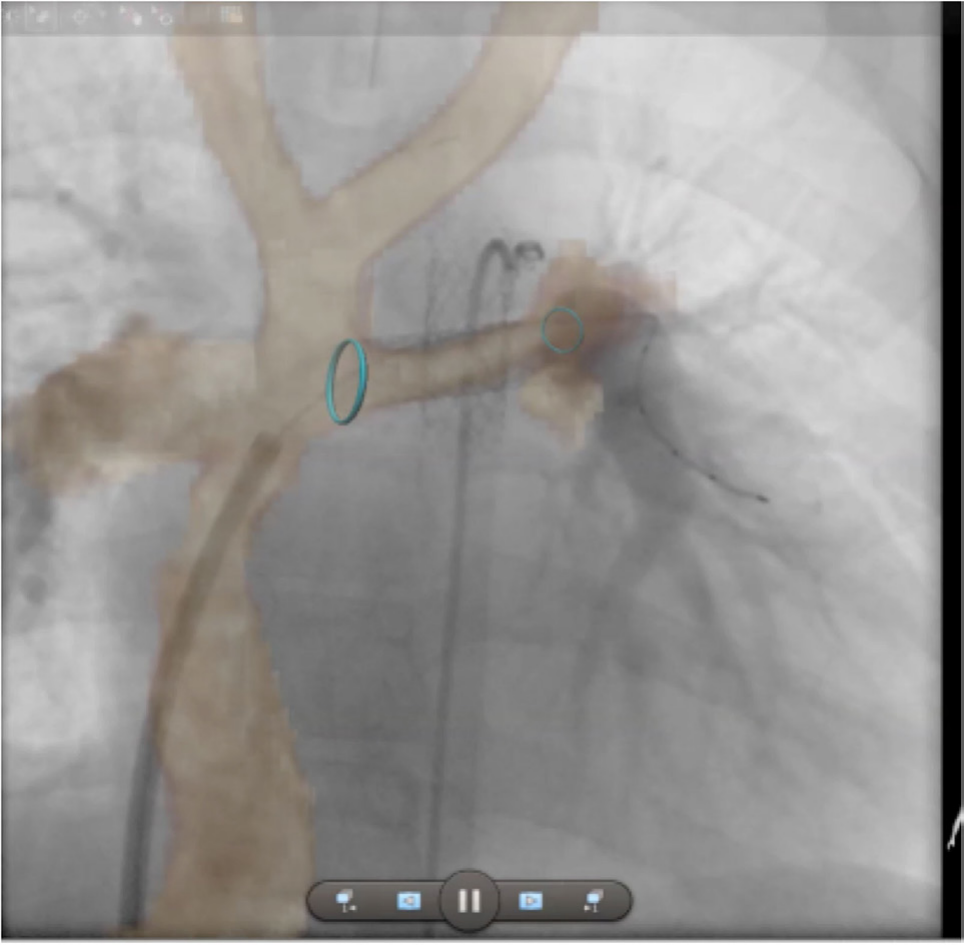
**Augmented fluoroscopy**. Three-dimensional whole heart MR images are fused with angiographic images to augment procedural guidance. In this case, the left pulmonary artery in a child with a Fontan circuit has been stented. The optimal angulation for imaging is predetermined using the MR dataset. Blue ring markers on the MRI dataset are used to guide the positioning of the stent. The images show good registration on the angiogram after stenting has taken place.

Finally, isotropic 3D whole heart datasets are particularly useful in CHD for interventional planning. MR angiography has already been shown to be accurate for planning cardiac catheterization procedures, with the 3D nature of the dataset being critical (43). 3D whole heart datasets, with the added advantage of gating, are well-suited to the production of 3D printed models to aid surgical planning (44, 45). More recently, clinicians have been fusing 3D whole heart datasets with conventional fluoroscopy angiography to augment procedural guidance. This approach can be used to minimize radiation, preplan angulations for fluoroscopic imaging, and reduce the required dose of iodinated contrast agent (Figure 11).

## Summary

ECG-gated respiratory-navigated 3D whole heart MRI has opened the door for isotropic submillimeter coronary imaging. Improved rest period delineation, image contrast agent use, and motion compensation, along with sequence acceleration and appropriate use of magnetization pre-pulses, have resulted in iterative improvements in image quality. The detail offered allows accurate segmental morphological analysis and has opened new avenues in research, teaching, and clinical diagnostics. A myriad of imaging applications, ranging from improved sequence planning during the course of the study, to volumetric analysis in complex ventricular geometries, and from 3D printing for surgical planning to computational modeling, have placed this sequence at the cornerstone of congenital cardiac MRI. The technique still requires diligence in planning, but current 3D whole heart images provide excellent quality overall and are often used to showcase image quality in clinical and commercial practice.

## Author Contributions

All the authors (GG, AT, MV, and TH) made substantial contributions to this review, worked on drafting and revising the work; approved the final version; and agreed to be held responsible for all aspects of the work.

## Conflict of Interest Statement

The authors declare that the research was conducted in the absence of any commercial or financial relationships that could be construed as a potential conflict of interest.

## Supplementary Material

Video S1 **A patient with functional single ventricle, who has undergone a Damus–Kaye–Stansel anastomosis and most recently a Fontan procedure**. This image was acquired using 3D whole heart techniques with gadofosveset trisodium as described in the text.

Video S2 **A patient with Scimitar syndrome**. This image was acquired using gadofosveset trisodium contrast and a 3D spoiled gradient echo 3D whole heart sequence.
